# Basic practices for gastrointestinal ultrasound

**DOI:** 10.1007/s10396-022-01236-0

**Published:** 2022-09-10

**Authors:** Mutsumi Nishida, Yuichi Hasegawa, Jiro Hata

**Affiliations:** 1 Diagnostic Center for Sonography, Hokkaido University Hospital, N14 W5, Kita-ku, Sapporo, 060-8648 Japan; 2 Department of Clinical Laboratory, Japanese Red Cross Narita Hospital, Narita, Japan; 3 Department of Laboratory Medicine (Endoscopy and Ultrasound), Kawasaki Medical School Hospital, Okayama, Japan

**Keywords:** Ultrasonography, Gastrointestine, Procedure, Diagnosis

## Abstract

The standard diagnostic modalities for gastrointestinal (GI) diseases have long been endoscopy and barium enema. Recently, trans-sectional imaging modalities, such as computed tomography and magnetic resonance imaging, have become increasingly utilized in daily practice. In transabdominal ultrasonography (US), the bowel sometimes interferes with the observation of abdominal organs. Additionally, the thin intestinal walls and internal gas can make structures difficult to identify. However, under optimal US equipment settings, with identification of the sonoanatomy and knowledge of the US findings of GI diseases, US can be used effectively to diagnose GI disorders. Thus, the efficacy of GIUS has been gradually recognized, and GIUS guidelines have been published by the World Federation for Ultrasound in Medicine and Biology and the European Federation of Societies for Ultrasound in Medicine and Biology. Following a systematic scanning method according to the sonoanatomy and precisely estimating the layered wall structures by employing color Doppler make diagnosing disease and evaluating the degree of inflammation possible. This review describes current GIUS practices from an equipment perspective, a procedure for systematic scanning, typical findings of the normal GI tract, and 10 diagnostic items in an attempt to help medical practitioners effectively perform GIUS and promote the use of GIUS globally.

## Introduction

The standard diagnostic modalities for gastrointestinal (GI) diseases have long been endoscopy and barium enema [1]. Recently, trans-sectional imaging modalities, such as computed tomography (CT) and magnetic resonance imaging (MRI), have become increasingly utilized [2, 3, 3–5]. In transabdominal ultrasonography (US), the GI can interfere with the observation of other abdominal organs. Additionally, the thin organ walls and internal gas make structures of the GI tract difficult to identify. However, frequent endoscopy is stressful for patients, and gaining endoscopic access can be difficult due to pain and stenotic lesions [6, 7]; the frequent use of CT increases the risk of carcinogenesis because of radiation [8–10]; and only a limited number of institutions have MRI, which is expensive and has low procedural throughput [2, 11].

Recently, the efficacy of transabdominal US has been reported [2, 12–31], and the World Federation for Ultrasound in Medicine and Biology [32] and the European Federation of Societies for Ultrasound in Medicine and Biology (EFSUM) have published guidelines on GIUS [1, 33–39]. However, in the EFSUM guidelines, the stomach is not included, and neither a precise scanning procedure nor typical normal GIUS findings are well documented. By systematic scanning [40] according to the anatomy and precisely estimating the wall layers, and using color Doppler [18, 41–44], properly diagnosing GI diseases becomes possible.

In an attempt to promote the use of GIUS by the GIUS study group in Japan, which began in 2006, this review provides precise descriptions of expanded scanning locations, optimal US equipment settings, a method for screening the entire intestine, including the stomach, a standard cutoff value for wall thickness, and useful indicators for identifying wall layers and diseases. Using this information, GIUS can be effectively used in daily practice.

## Optimal US equipment settings and patient preparation

A convex probe with a central frequency of 3.5–6 MHz and a linear probe with a frequency of 7.5–12 MHz can be used. If no deep attenuation occurs, a higher frequency probe is recommended [45–47].

Because most of the intestine is in a shallow location, the imaging depth is set to 8 cm or less according to the target instead of the default 15 cm setting, which is mainly used for the liver. To maximize the target appropriately, the focus point is located just below the target. A lower gain setting is preferable to see thin intestinal walls that may contain some amount of gas, and a narrower dynamic range makes it easier to identify layers in thin walls with harmonic imaging [48]. These parameters can be changed quickly by creating a preset button named “Intestine” or “Bowel”. To delineate longer GI tract segments, panoramic imaging and a wider view are helpful for better orientation [49, 50].

Fasting over 4 h is recommended for patients [33]. An overnight fast (> 8 h) will improve visibility and minimize the meal effect. However, the full stomach and full bladder techniques are useful for seeing the stomach and duodenum, and rectum, respectively [51–56].

Color Doppler imaging can be performed concomitantly, with the color Doppler gain adjusted to eliminate noise and maximize sensitivity. The color Doppler frequency is set from 2–7 MHz, and the pulse repetition frequency is set from 4–10 cm/sec; these frequencies can be adjusted according to the type of probe and the depth of the target. The wall filter is set from 3–4. An increased Doppler signal is defined as a spotty to linear color Doppler signal in the mucosa and submucosa. The blood flow signal is semiquantitatively classified as grade 0–3 [18, 41–44, 57].

## Systematic scanning method

**Fig. 1:**
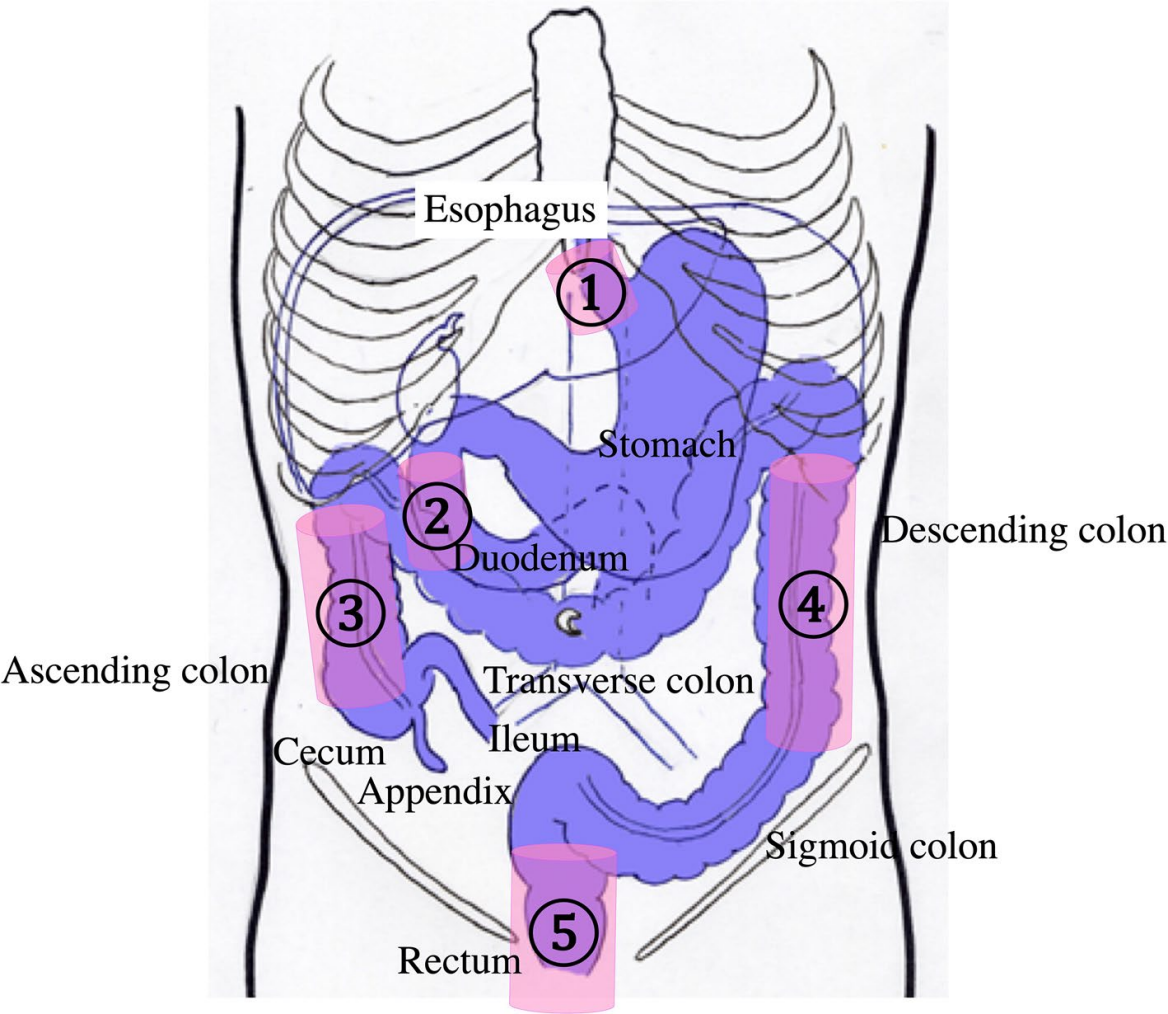
**Fixed parts of the bowel in the abdominal cavity** (pink columns). The abdominal esophagus is fixed by the esophageal hiatus ①. The second portion of the duodenum ②, the ascending colon ③, the descending colon ④, and the rectum ⑤ are fixed to the retroperitoneum

Many parts of the intestine are not fixed and meander as tubular structures. Orientation is made more difficult by the lack of landmark vessels. However, systematic scanning according to the anatomy increases the likelihood of detecting and identifying the location of lesions [40]. When performing systematic scanning, it is important to note the fixed locations of the GI tract. These include the esophageal hiatus, the second duodenal portion, the ascending colon, the descending colon, and the rectum, which are fixed by the retroperitoneum (Fig. 1). Unfixed parts are detectable by tracing from fixed parts, such as the transverse and sigmoid colon. The GI tract is sequentially assessed, including the esophagus, stomach, duodenum, colon, jejunum, and ileum. Essentially, each part of the GI tract must be scanned on two perpendicular planes to avoid an oversight, avoid artifacts, and confirm the presence of a disorder.

Detailed systematic scanning methods are described below.

**Fig. 2:**
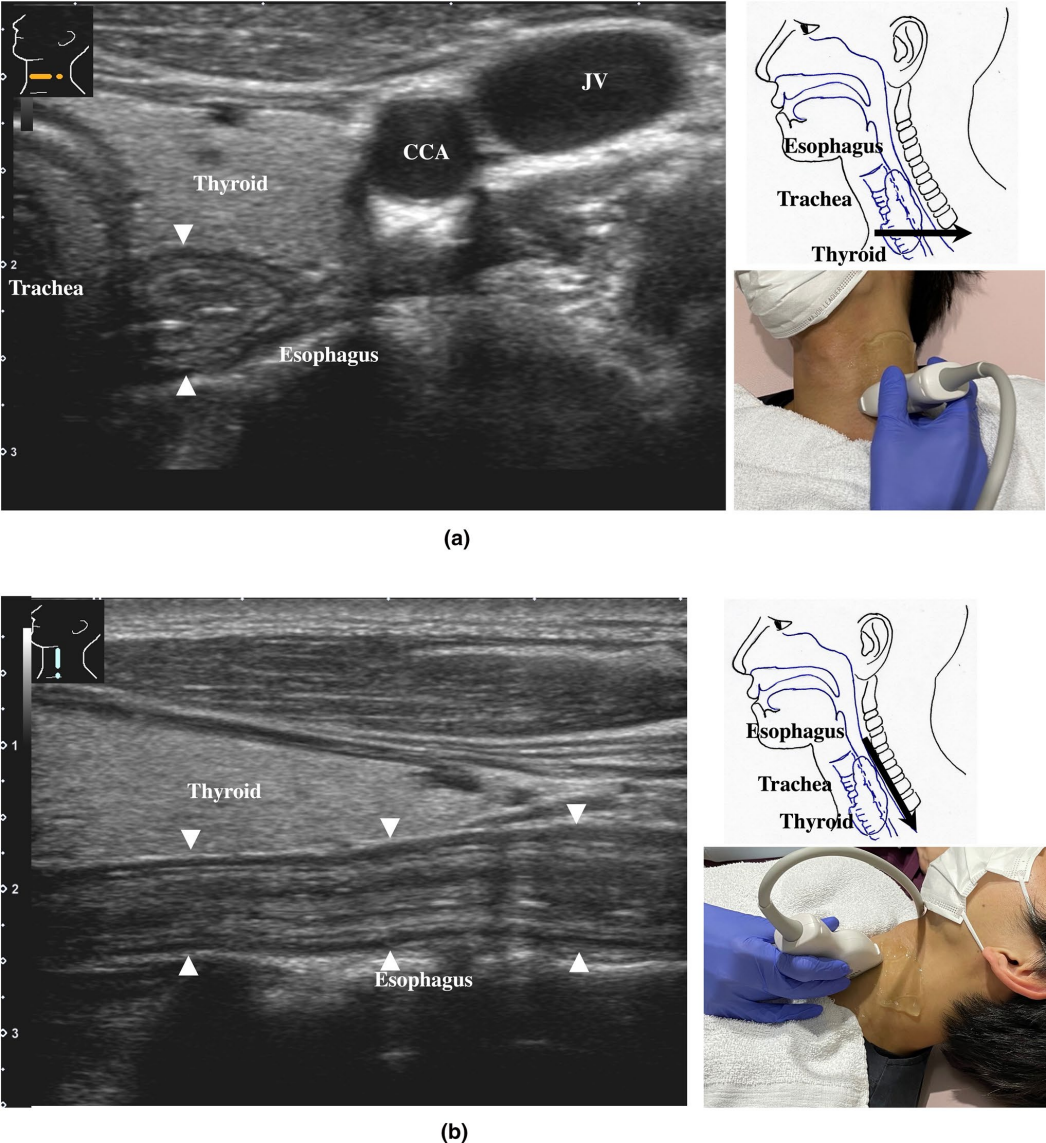
**Cervical esophagus** The cervical esophagus is located dorsal to the left thyroid lobe (**a**). Turning the probe 90 ° provides a longitudinal view of the esophagus (**b**)

1. The esophageal orifice is difficult to observe with endoscopy but easy to observe with US. The cervical esophagus is in a shallow location, so a high-frequency probe (more than 7.5 MHz) can be utilized. The esophagus runs straight on the dorsal side of the left thyroid lobe (Fig. 2). By the swallowing of saliva, constriction and movement of contents can be observed, and the cervical esophagus can be easily identified. The thoracic esophagus is more difficult to observe because it is located inside the chest.

**Fig. 3:**
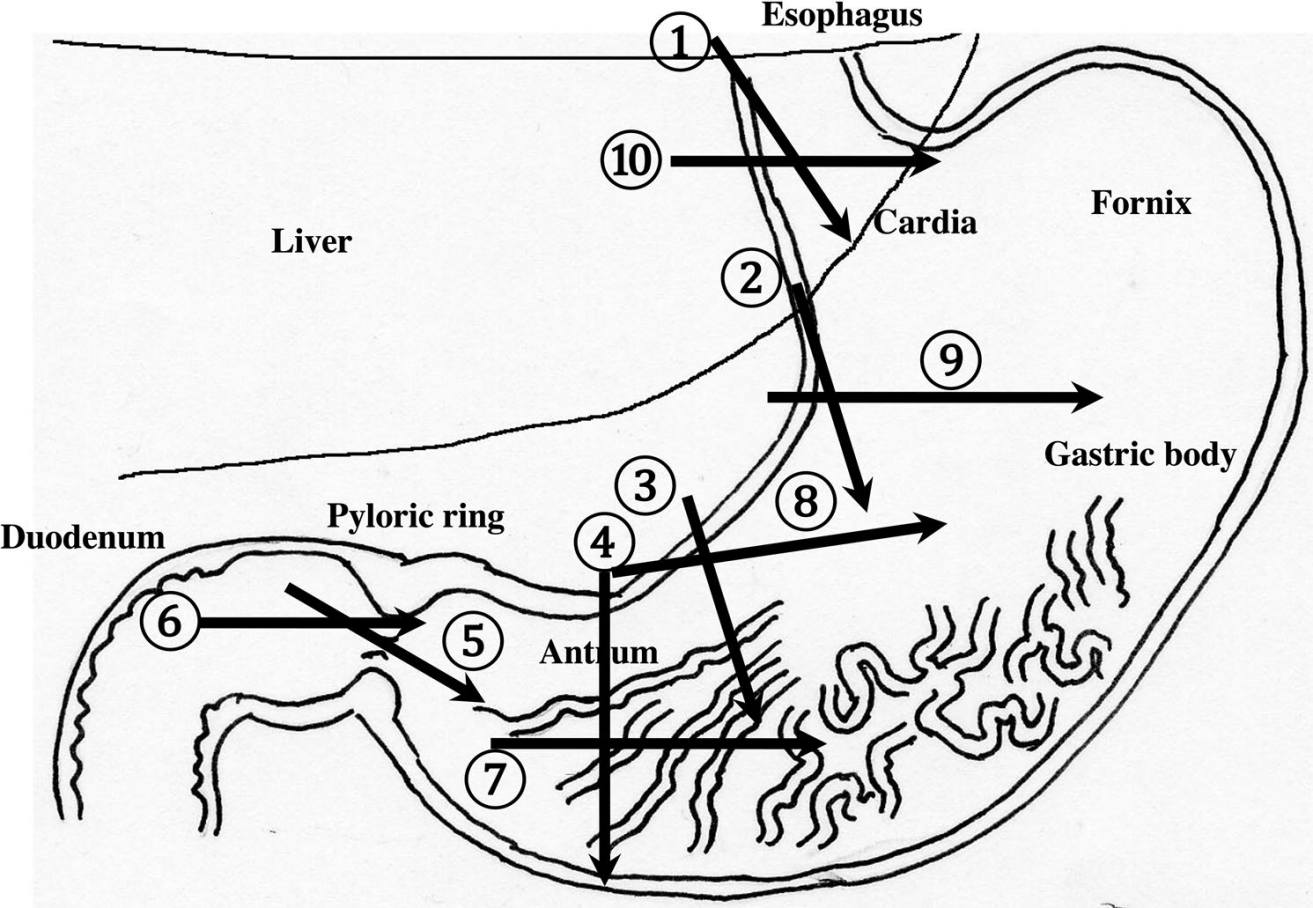
**Systematic scanning procedure for the upper GI tract** The recommended schematic procedure is shown. Starting from the abdominal esophagus, scanning proceeds to the pylorus and the duodenal bulb. The probe is moved in a reverse “C” shape. Then, the probe is turned 90 ° and returned from the duodenal bulb to the abdominal esophagus, retracing the reverse “C” curve

The recommended procedures for the upper GI tract are shown in Fig. 3. Routine screening usually covers the area from the abdominal esophagus to the duodenal bulb, balancing the prevalence of disease with the examination time.

**Fig. 4:**
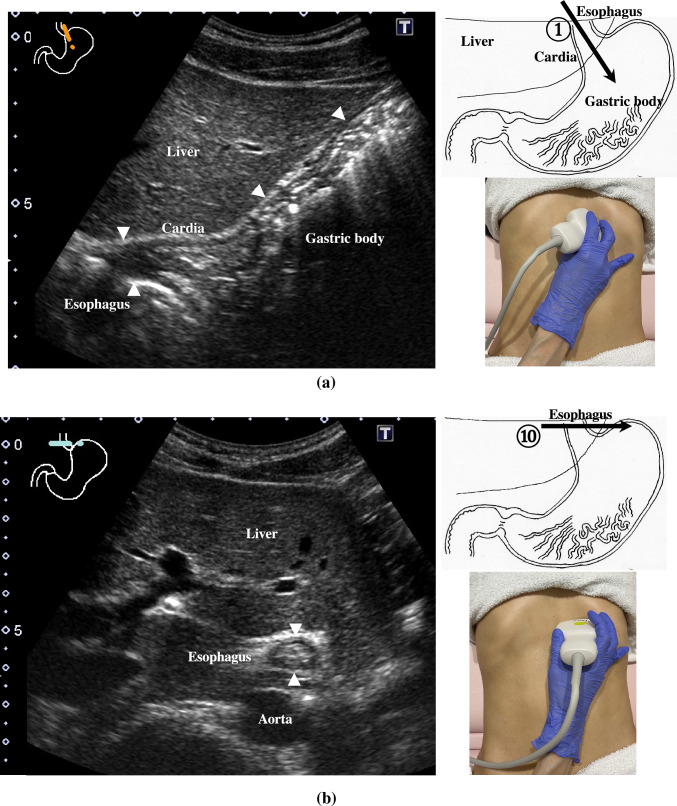
**Abdominal esophagus** The abdominal esophagus can be seen between the left lobe of the liver and aorta by aiming the probe up toward the upper left epigastrium (**a**). This is the starting point of the upper GI tract scan. The short axis of the esophagus can be seen as a ring-like structure on axial scanning with the probe aimed up toward the epigastrium (**b**)

**Fig. 5:**
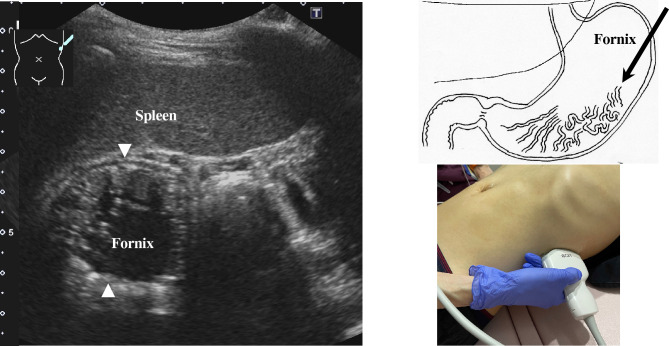
**Gastric fornix** The fornix is observed by a left intercostal scan behind the spleen. The spleen can serve as a good acoustic window

**Fig. 6:**
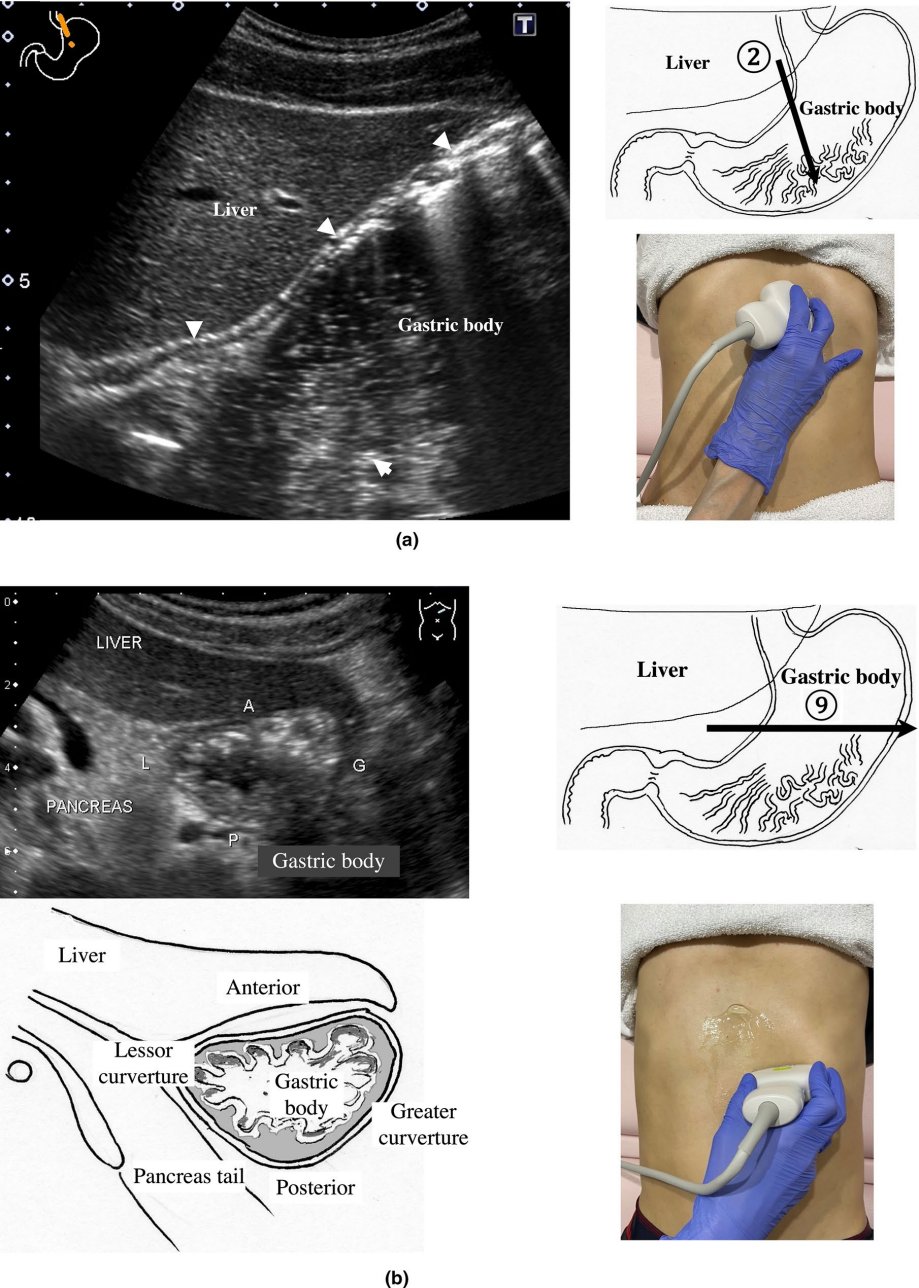
**Gastric body** Following the continuity of the muscularis propria (hypoechogenic wall layer) from the abdominal esophagus, the gastric body can be observed as a beak sign by moving the probe caudally (**a**). Deep inhalation stretches the gastric body and makes it easy to recognize. Axial scanning of the gastric body shows the short axis of the gastric body, which is helpful for anatomical orientation (**b**). The left lateral decubitus position is effective when the gastric body is indistinct, and this body position is useful for the full stomach method

**Fig. 7:**
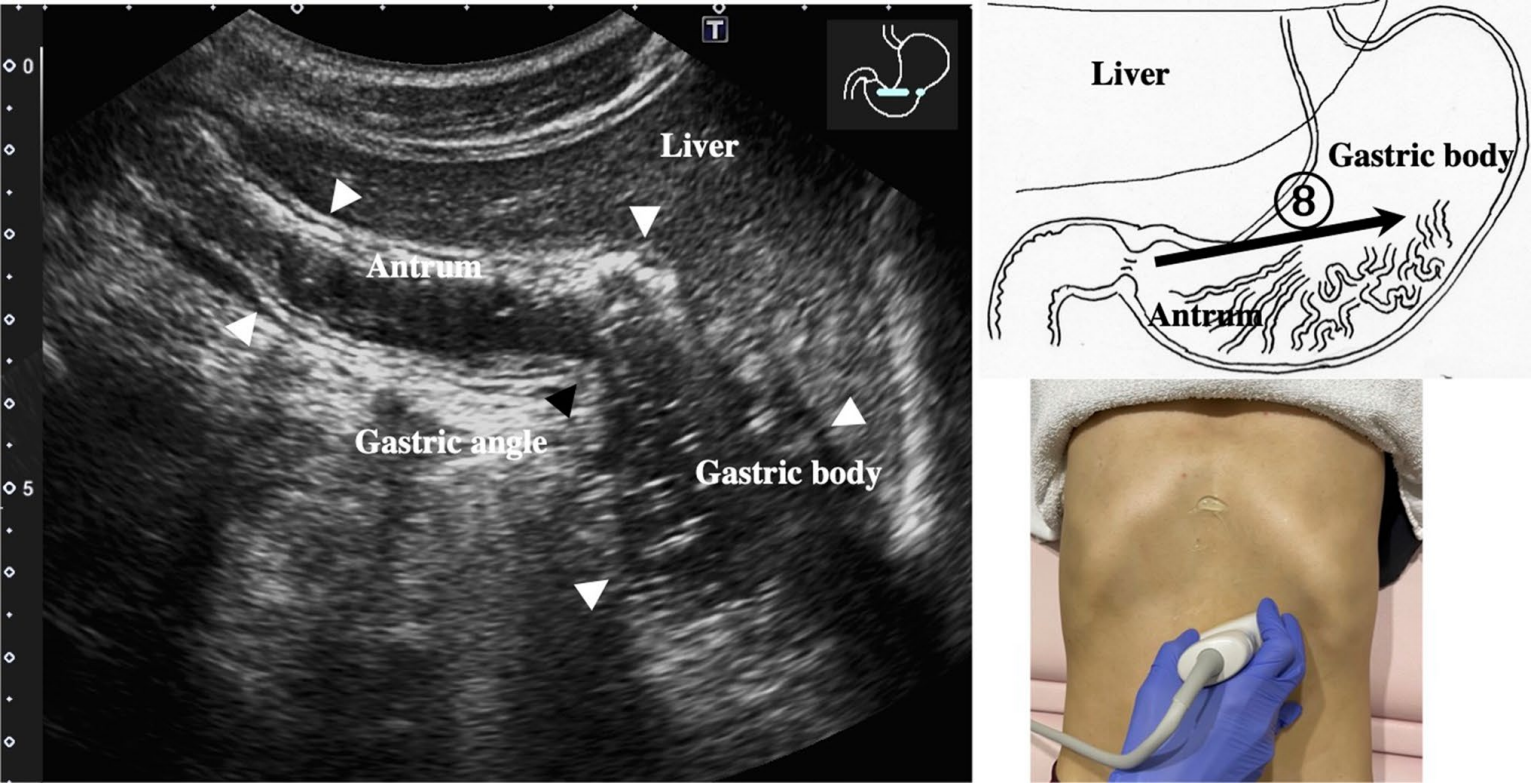
**Gastric angle** Moving caudally from the position shown in Fig. 6, scanning proceeds to the right by parallel translation, from the gastric body through a slightly constricted angle (black arrowhead) and continuing to the antrum

**Fig. 8:**
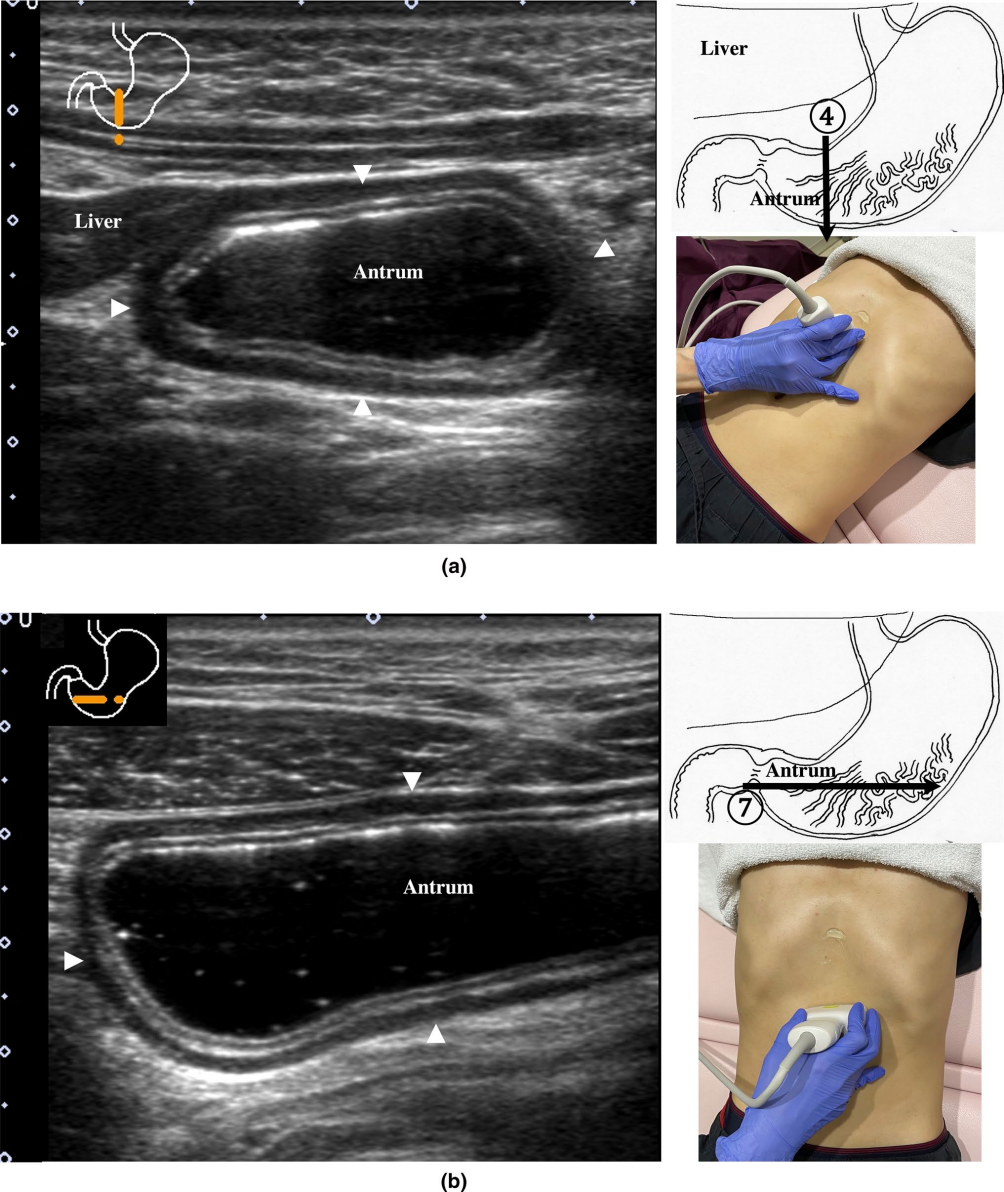
**Gastric antrum** The gastric antrum can be observed easily, usually without interference from the ribs; it is in a shallow location in the abdominal cavity. A high-frequency probe (more than 7.5 MHz) with the full stomach method is employed. Sagittal view (**a**), longitudinal view [65] (**b**)

**Fig. 9:**
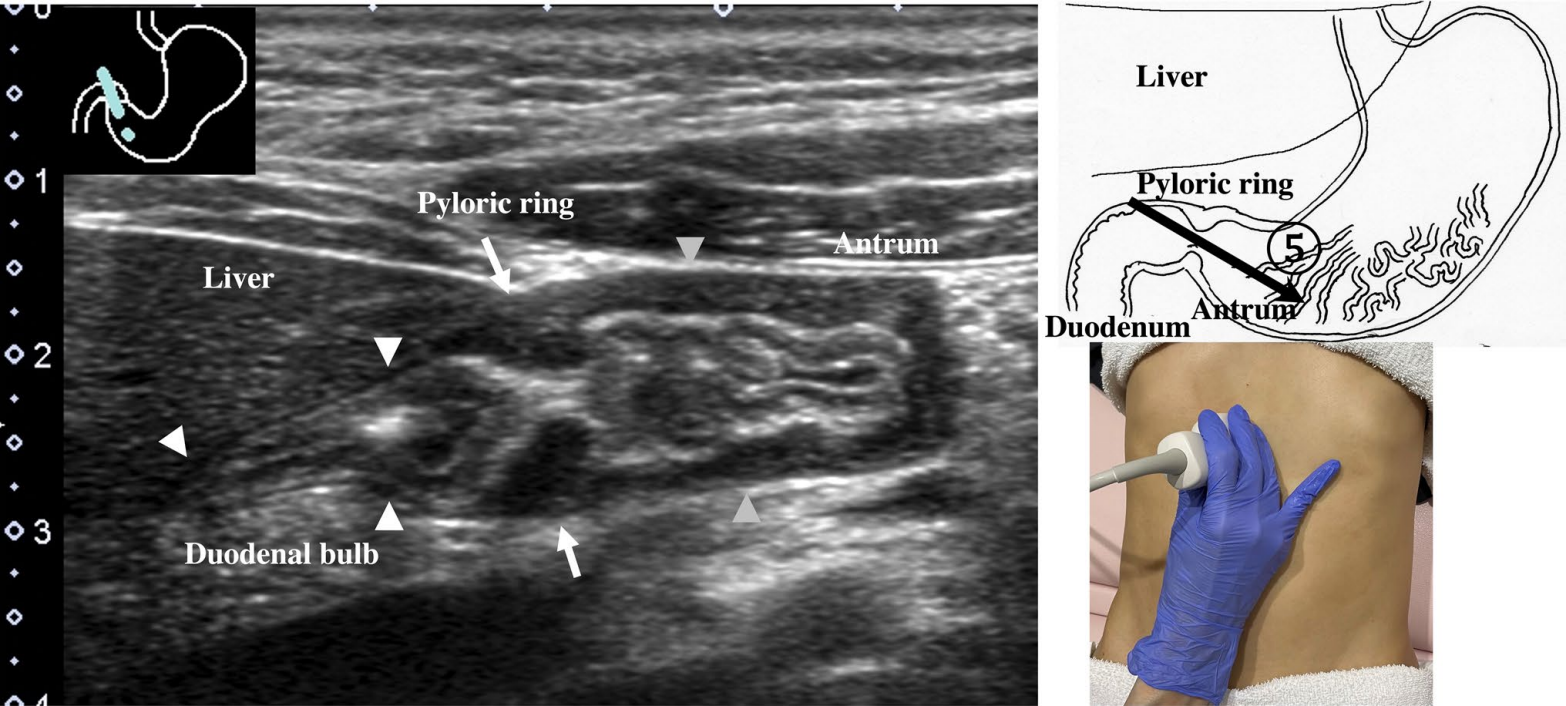
**Pyloric ring and duodenal bulb** From the sagittal plane of the antrum (gray arrow heads), moving parallel to the right, the probe is rotated about 30 ° counterclockwise to identify the pyloric ring; continuing along the wall reveals the duodenal bulb. The muscularis propria is the thickest in the pyloric ring due to the pyloric sphincter (arrow). The duodenal wall is thinner than the gastric wall (white arrowhead)

**Fig. 10:**
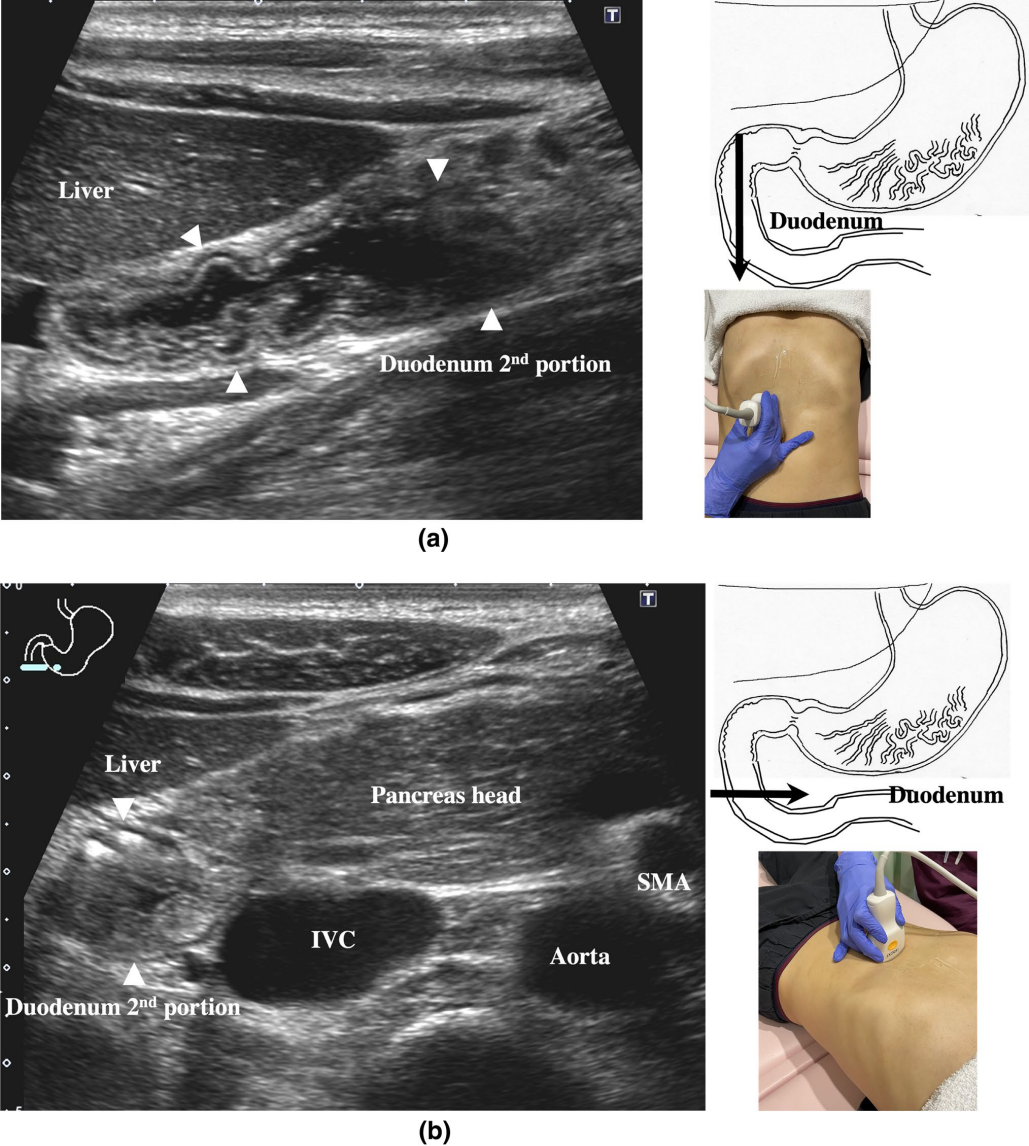
**Second duodenal portion** Longitudinal view of the second duodenal portion. From the duodenal bulb, the second duodenal portion forms a “C”-shaped curve around the pancreatic head. Sagittal view (**a**), axial view (**b**). The full stomach method is effective for identifying the lumen of the duodenum

**Fig. 11:**
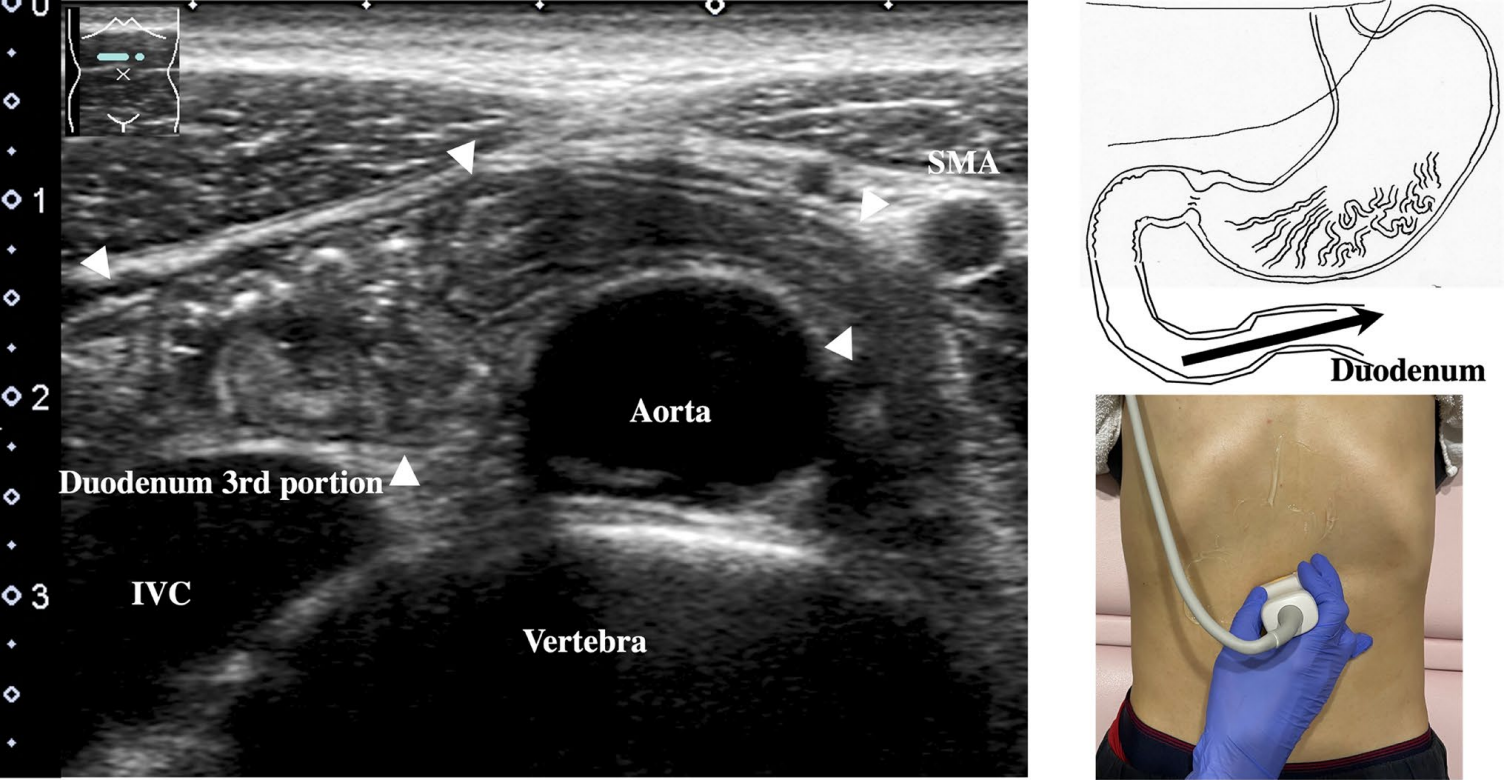
**Third duodenal portion** Longitudinal view of the third duodenal portion. From the second duodenal portion, an axial scan is performed on the midline, revealing the third duodenal portion between the abdominal aorta and superior mesenteric artery (SMA). If following the continuity from the second duodenal portion to the third portion is difficult, the probe can be turned 90 ° to allow identification of the short axis of the third portion between the abdominal aorta and SMA

2. The abdominal esophagus is located between the left liver lobe and the abdominal aorta, surrounded by the crura of diaphragm (Fig. 4).
3. The stomach can be traced from the abdominal esophagus starting the cardia through the pyloric ring. The fornix can also be observed from a left intercostal approach at the caudal side of the spleen (Fig. 5). The gastric body is located on the dorsal side of the left lobe of the liver (Fig. 6a). Taking a deep breath stretches and pushes the gastric body to the caudal side, allowing the wall of the gastric body to be observed easily. On axial scanning of the gastric body, the anterior wall, posterior wall, lesser curvature, and greater curvature can be identified (Fig. 6b). Moving the probe caudally on the right side of the patient reveals constriction of the gastric body, which continues to narrow with further movement toward the antrum (Fig. 7).
4. A sagittal scan of the epigastrium shows the short axis of the antrum on the caudal side of the left liver lobe (Fig. 8a). Alternatively, from the gastric body (Fig. 6), the antrum can be easily observed by moving the probe in a reverse “C” shape. The antrum is in a shallow location in the abdominal cavity close to the abdominal wall. A high-frequency probe is effective for observing the wall layers. The long axis of the antrum can be observed by rotating the probe 90 ° (Fig. 8b).
5. From the short axis of the antrum, parallel translation toward the right side reveals the pyloric sphincter; then, after rotating the probe 30 ° counterclockwise, the tract can be traced to the duodenal bulb wall (Fig. 9). The duodenal wall is thinner than the gastric wall and may be difficult to identify. The duodenal wall is located cranial to the antrum and on the left side of the gallbladder.
6. The second portion of the duodenum can be scanned by tracing from the duodenal bulb (Fig. 10a). The second portion runs in a “C” shape surrounding the pancreatic head. The short axis of the second portion is located next to the pancreas head (Fig. 10b).
7. The third portion of the duodenum runs between the abdominal aorta and the superior mesenteric artery (SMA) (Fig. 11). A sagittal scan on the midline can be used to identify the third portion between the SMA and abdominal aorta.

**Fig. 12:**
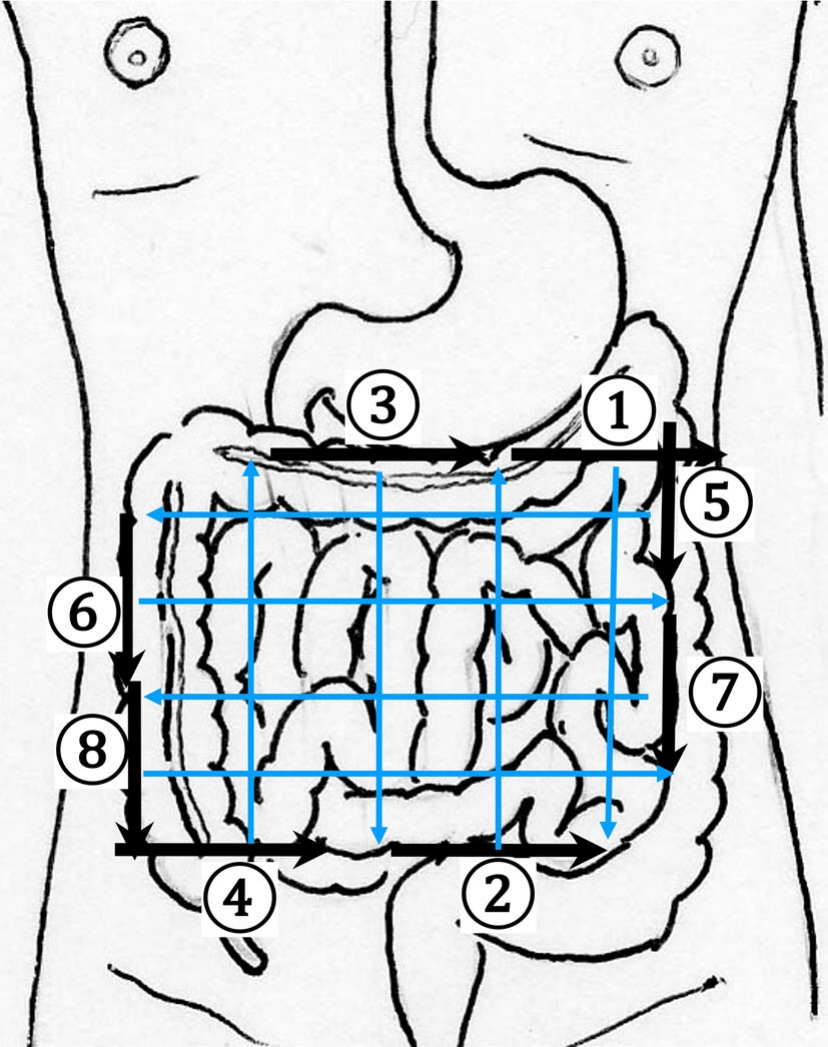
**Comprehensive scanning procedure for the jejunum and the ileum** Systematic scanning is difficult to perform for the small intestine since it is attached to the intestinal membrane. Comprehensive scanning is recommended. Specifically, because the intestinal membrane is attached to the retroperitoneum, from the upper left abdomen to the lower right abdomen, the entire small intestine can be scanned by parallel translation of the probe from two directions

The entire small intestine (jejunum and ileum) is difficult to trace because of its mesenteric connections and high mobility. As systematic scanning is difficult, a comprehensive procedure is needed (Fig. 12). The scan starts from the upper left abdomen on the axial plane and progresses to the caudal end of the abdominal cavity, followed by a gradual parallel slide to the right, and then a return to the cranial end. The scan is repeated until the lower right abdomen is reached. Then, the probe is rotated to the sagittal plane, and scanning is performed starting from the upper left abdomen and progressing to the right. The probe is gradually moved via parallel translation to the caudal side and is then returned to the left abdomen. In the same manner, the probe is gradually translated to the caudal side and to the right until the lower right abdomen is reached. As the mesentery is fixed to the retroperitoneum, the jejunum is approximately located in the upper left abdomen, while the ileum is approximately located in the lower right abdomen.

**Fig. 13:**
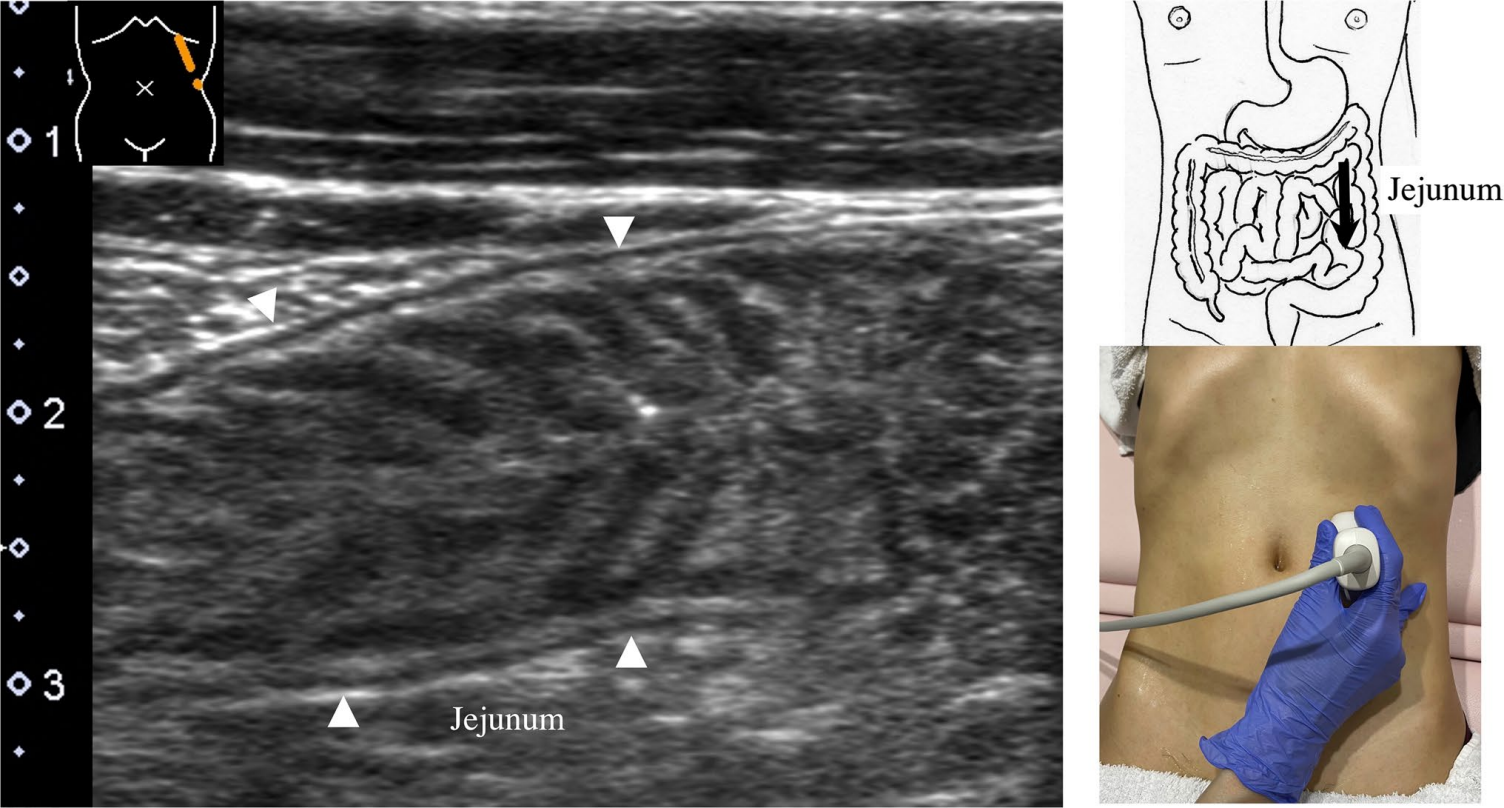
**Jejunum** Longitudinal view of the jejunum. The intestinal membrane is attached to the retroperitoneum from the upper left to the lower right of the abdomen. The jejunum is approximately located in the upper left abdomen and has large, dense Kerckring folds

**Fig. 14:**
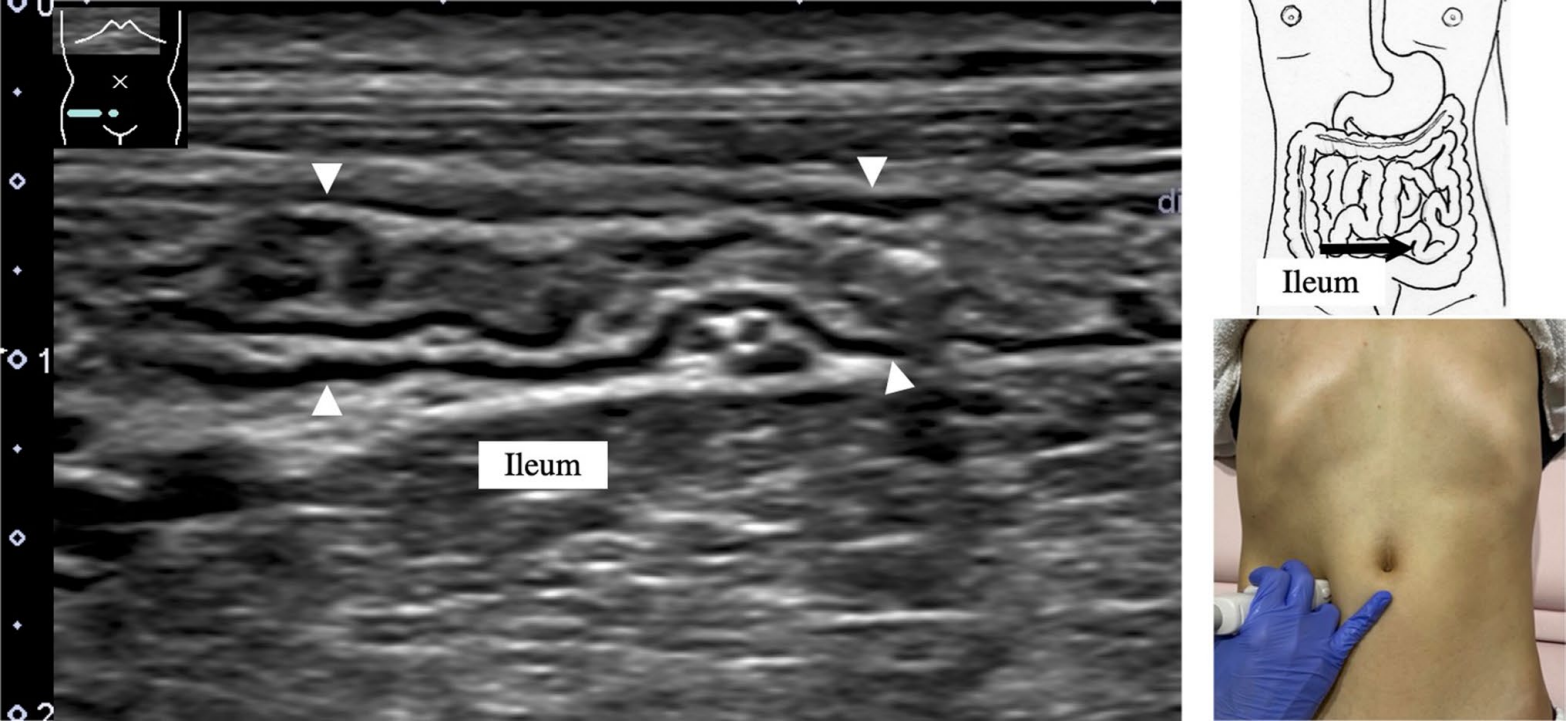
**Ileum** Longitudinal view of the ileum. The ileum is approximately located in the lower right abdomen, with small, sparse Kerckring folds

**Fig. 15:**
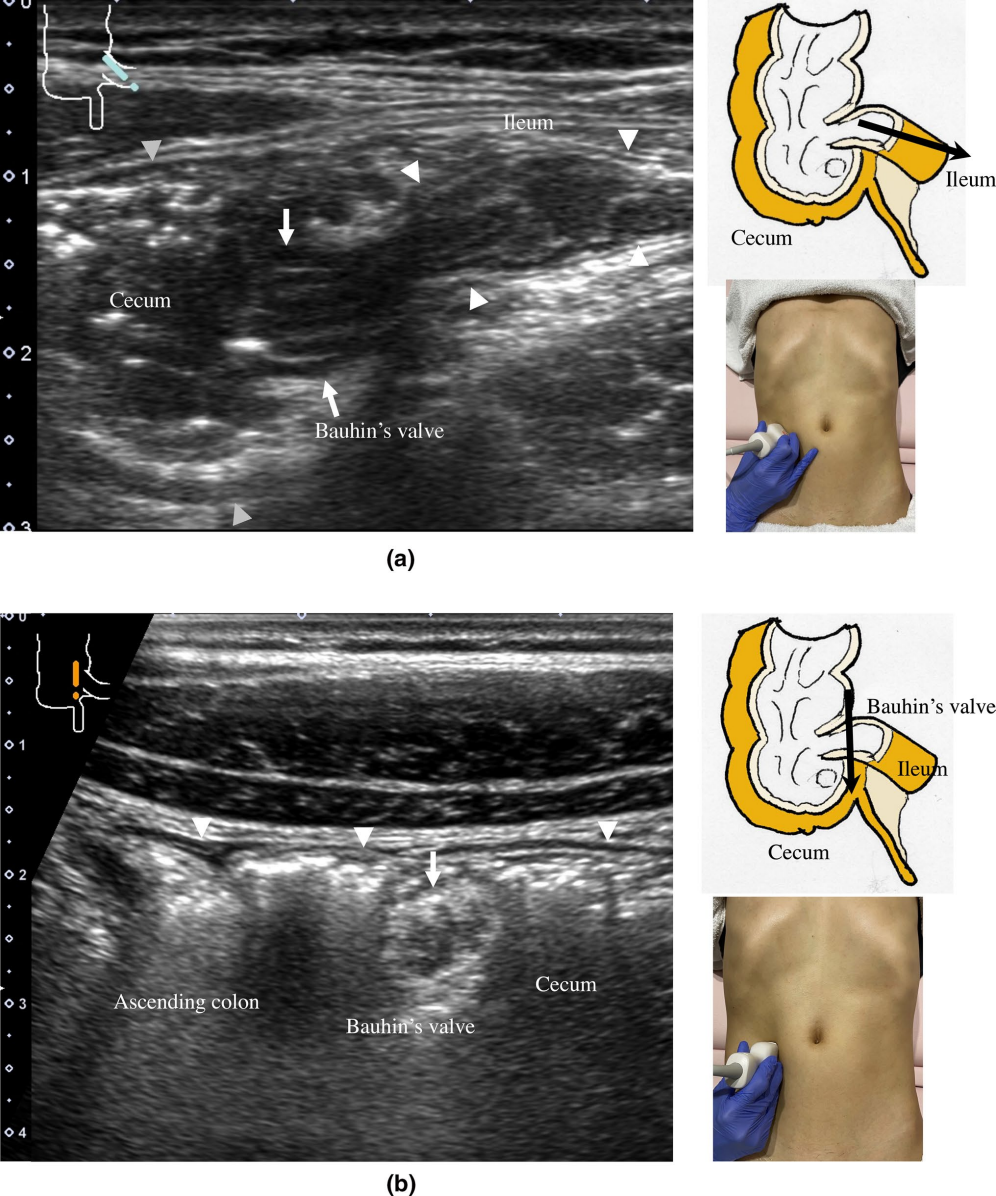
**Ileocecum** Longitudinal view of the terminal ileum (white arrowheads) (**a**). Bauhin’s valve (arrow) and the short axis of the cecum (gray arrowheads). The terminal ileum connects perpendicularly to the cecum, exhibiting the so-called mushroom sign. Short-axis view of Bauhin’s valve (arrow), longitudinal view of the cecum and the ascending colon (white arrow heads) (**b**)

8. The jejunum has large and dense Kerckring folds (Fig. 13) and a high level of peristalsis, while the ileum has small and sparse folds (Fig. 14). The terminal ileum runs in front of the external iliac artery/vein and iliopsoas muscle and continues to Bauhin’s valve, appearing as a “mushroom sign” (Fig. 15a). Bauhin’s valve is identified vertically connecting the large bowel to the cecum on the sagittal plane (Fig. 15b, arrow).

**Fig. 16:**
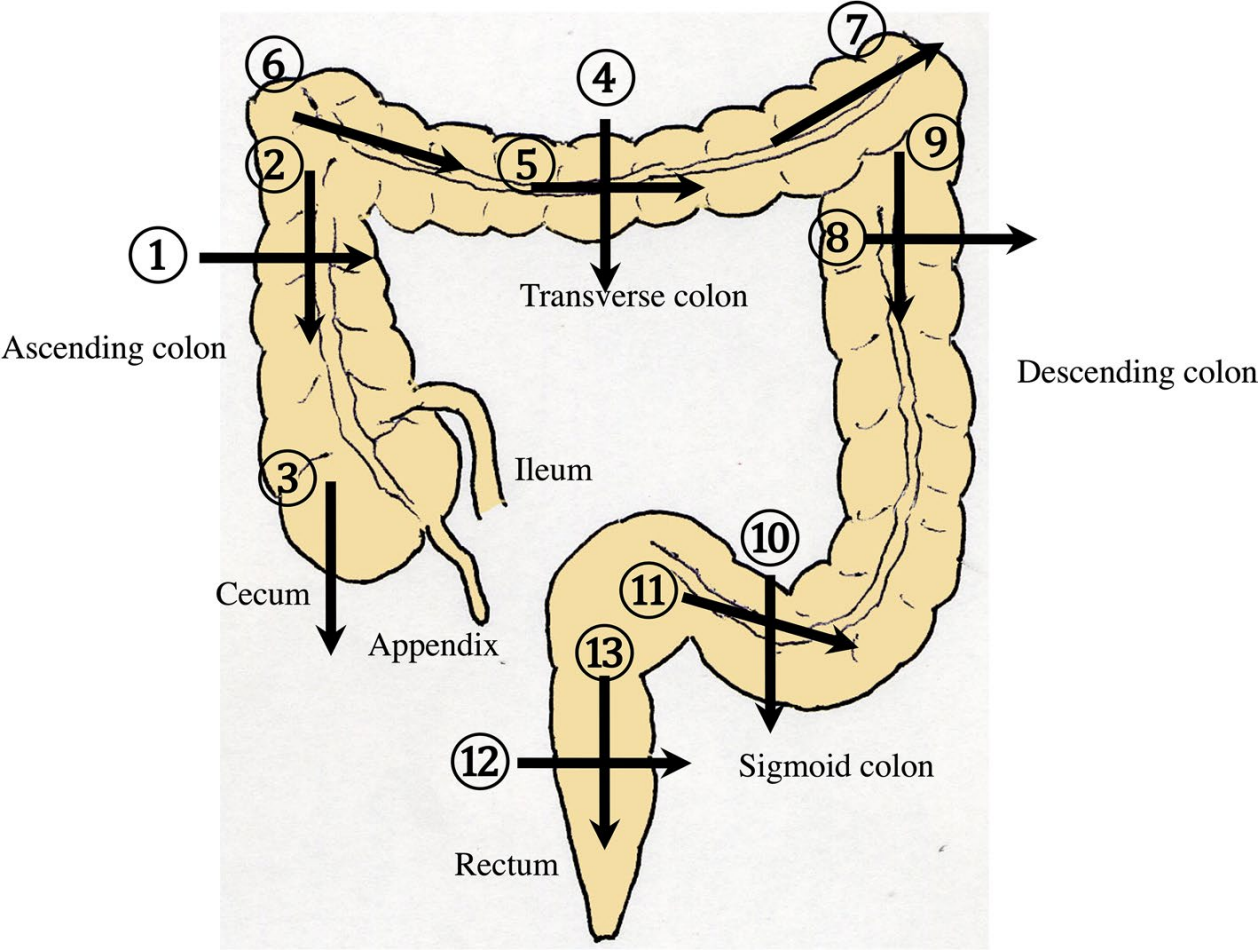
**Systematic scanning procedure for the colon** The recommended schematic procedure is shown. The colon and rectum are sequentially assessed, starting from the axial view of the ascending colon. To identify the ascending colon, its location needs to be confirmed; it is located in the outermost and backmost area on the right side of the abdominal cavity. Then, scanning proceeds to the cecum, identified by the blind end, and the terminal ileum and Bauhin’s valve can be identified. Then, scanning continues by returning the probe to the hepatic flexure (HF). The transverse colon can be identified by a sagittal scan on the midline caudal to the gastric antrum. From the midline, the transverse colon can be traced to the HF and then to the splenic flexure (SPF). As the SPF is located deeper on the dorsal side, deep inhalation or the right decubitus position may be required to observe it. The descending colon is identified on the left-back side. Finally, the colon is traced from the sigmoid colon to the rectum, which is visualized through the urinary bladder

**Fig. 17:**
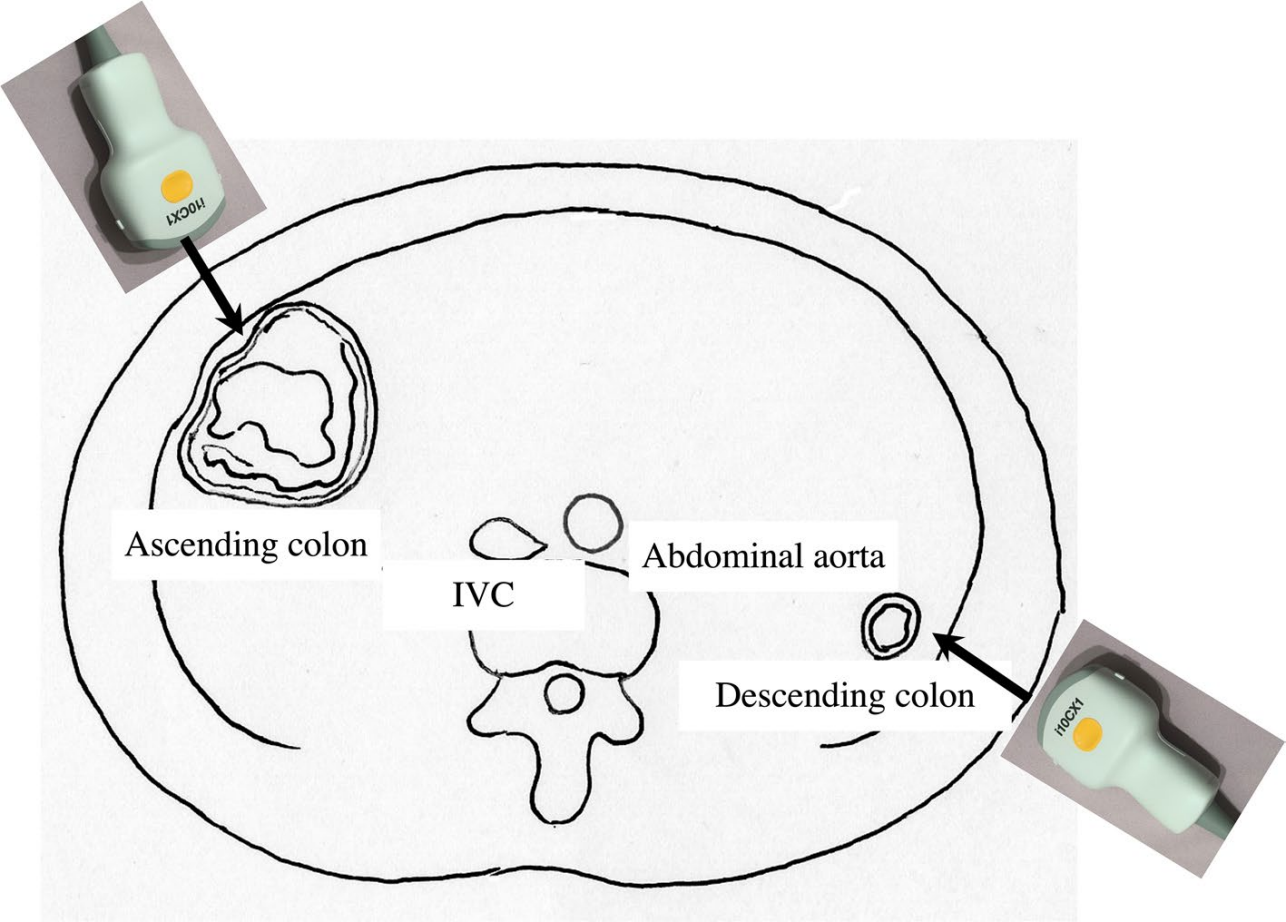
**Optimal approach angle to the ascending and descending colon** Schematic view of the axial plane of the abdomen. The ascending colon is observed using a ventral approach, while the descending colon can effectively be observed using a dorsal approach

The recommended procedure for the colon is shown in Fig. 16 [58]. The probe approach angles for the ascending colon and the descending colon are shown in Fig. 17. The ascending colon is in a relatively shallow location; in contrast, the descending colon [especially the splenic flexure (SPF)] is located deep on the dorsal side. Detailed systematic scanning methods for sequentially assessing the colon and the rectum are described below.

**Fig. 18:**
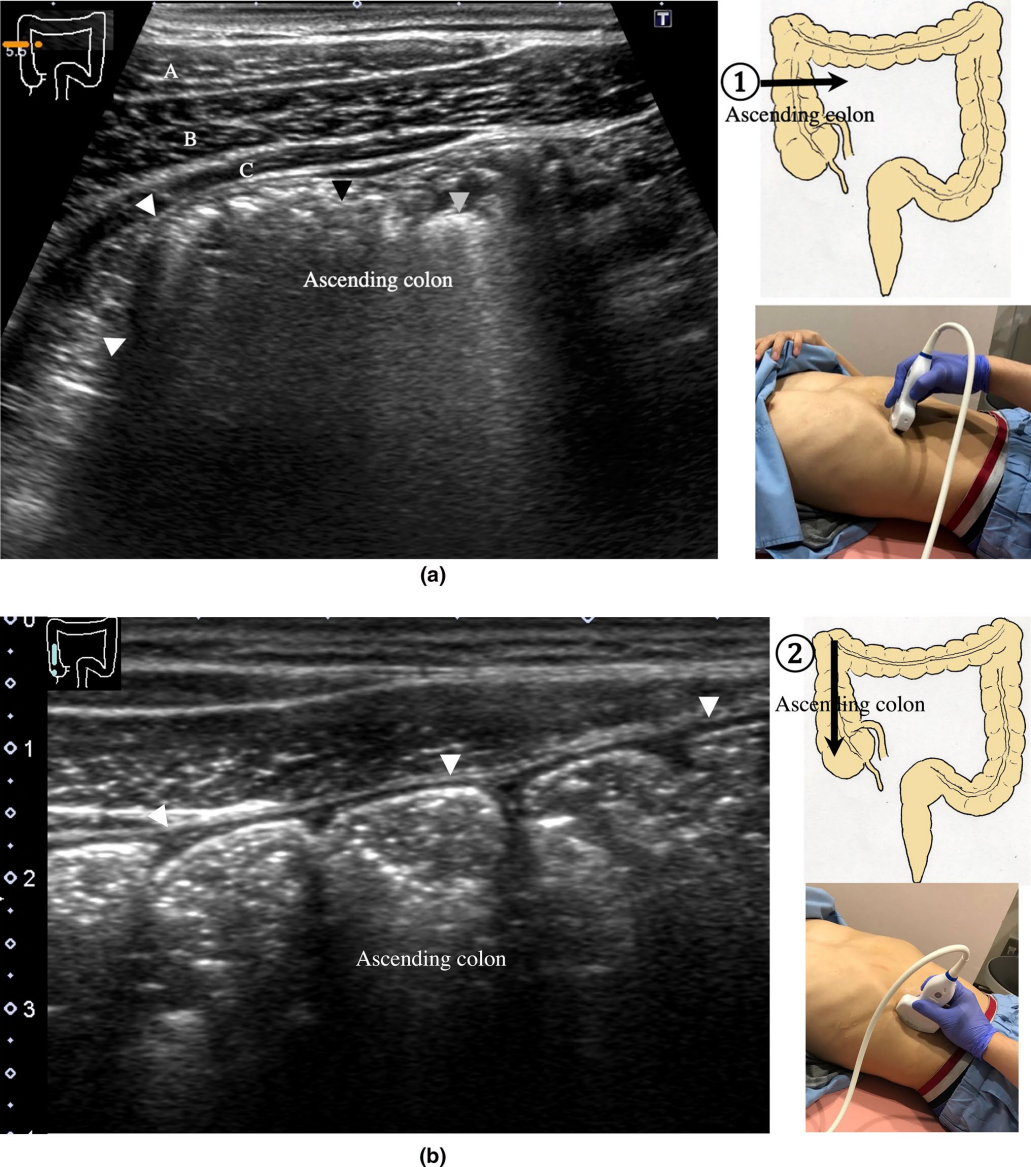
**Ascending colon** Short-axis view of the ascending colon (**a**). A thin wall, stool with an acoustic shadow (black arrowhead), and air with multiple reflections (gray arrowhead) are shown. The external oblique muscle (A), internal oblique muscle (B), and transverse abdominal muscle (C) can be identified in front of the ascending colon in the abdominal wall. Long-axis view of the ascending colon (**b**). Haustra are shown. The posterior wall is hardly visible

**Fig. 19:**
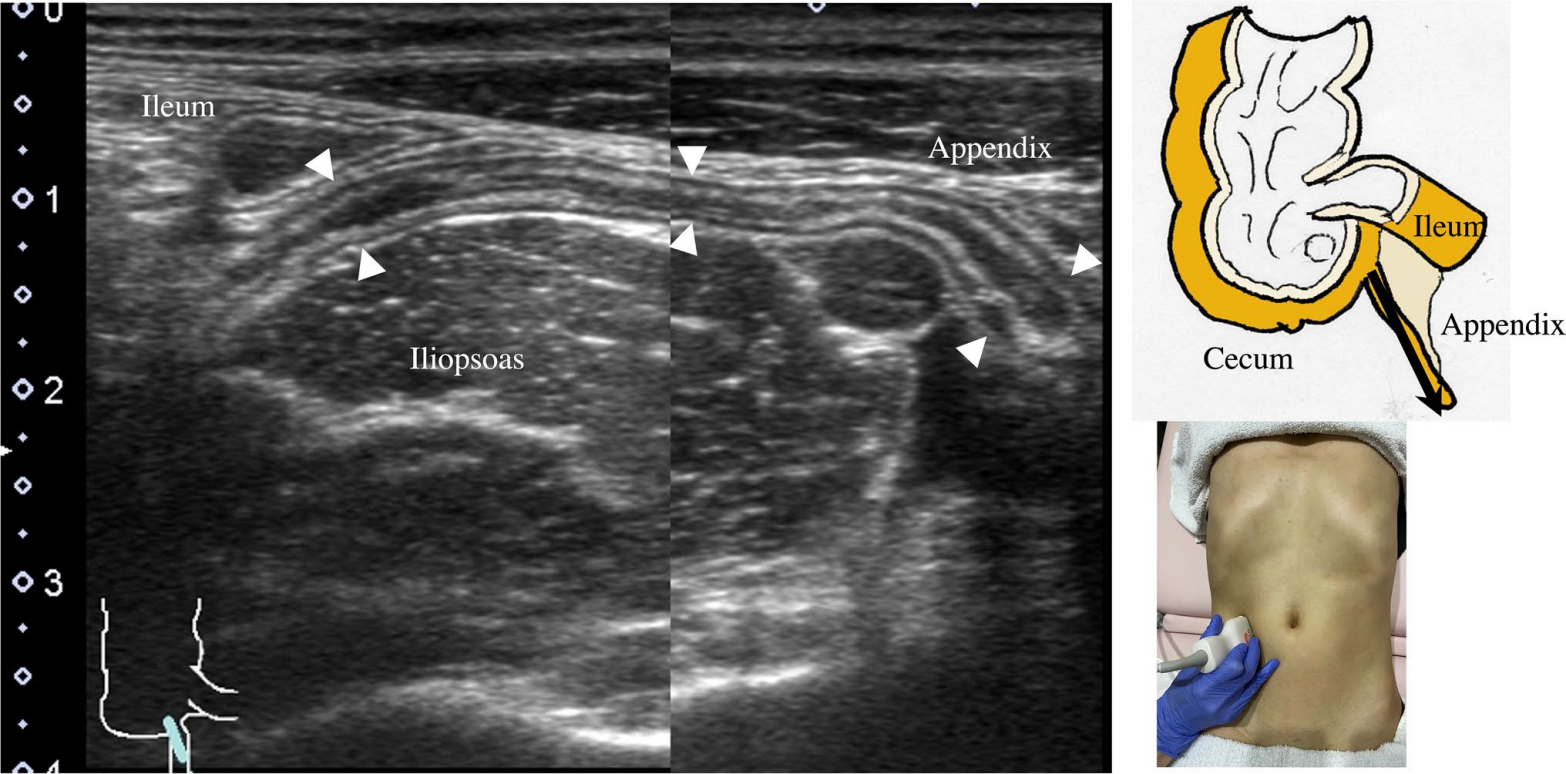
**Appendix Longitudinal** view of the appendix in front of the iliopsoas muscle and external iliac artery and behind the short axis of the terminal ileum. The appendix has a blind end and is usually located in the pelvic cavity

**Fig. 20:**
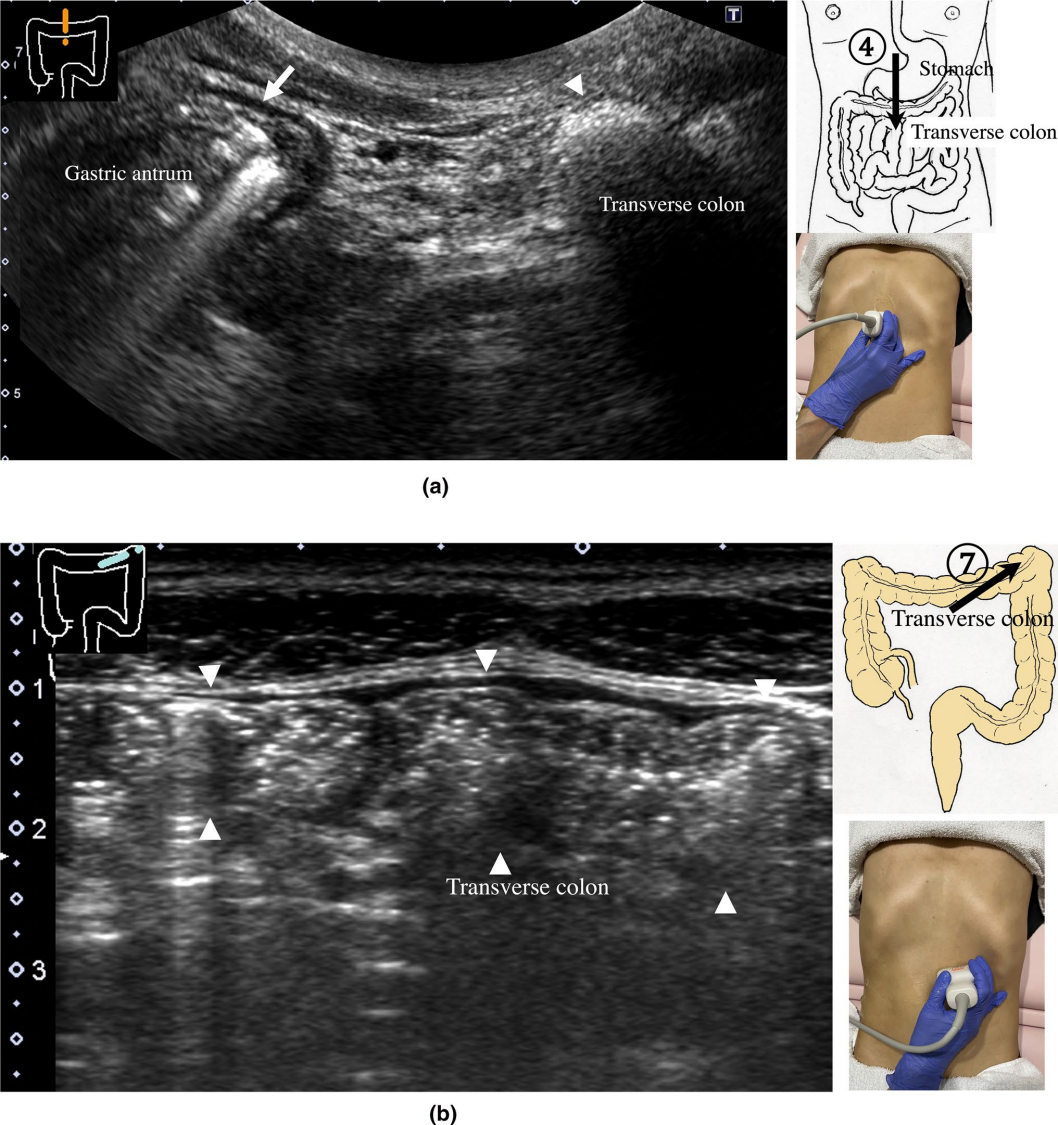
**Transverse colon** Sagittal scan on the midline shows the antrum (arrow) and transverse colon (arrowhead) on the caudal side (**a**). Transverse scan shows a longitudinal view of the transverse colon (**b**)

**Fig. 21:**
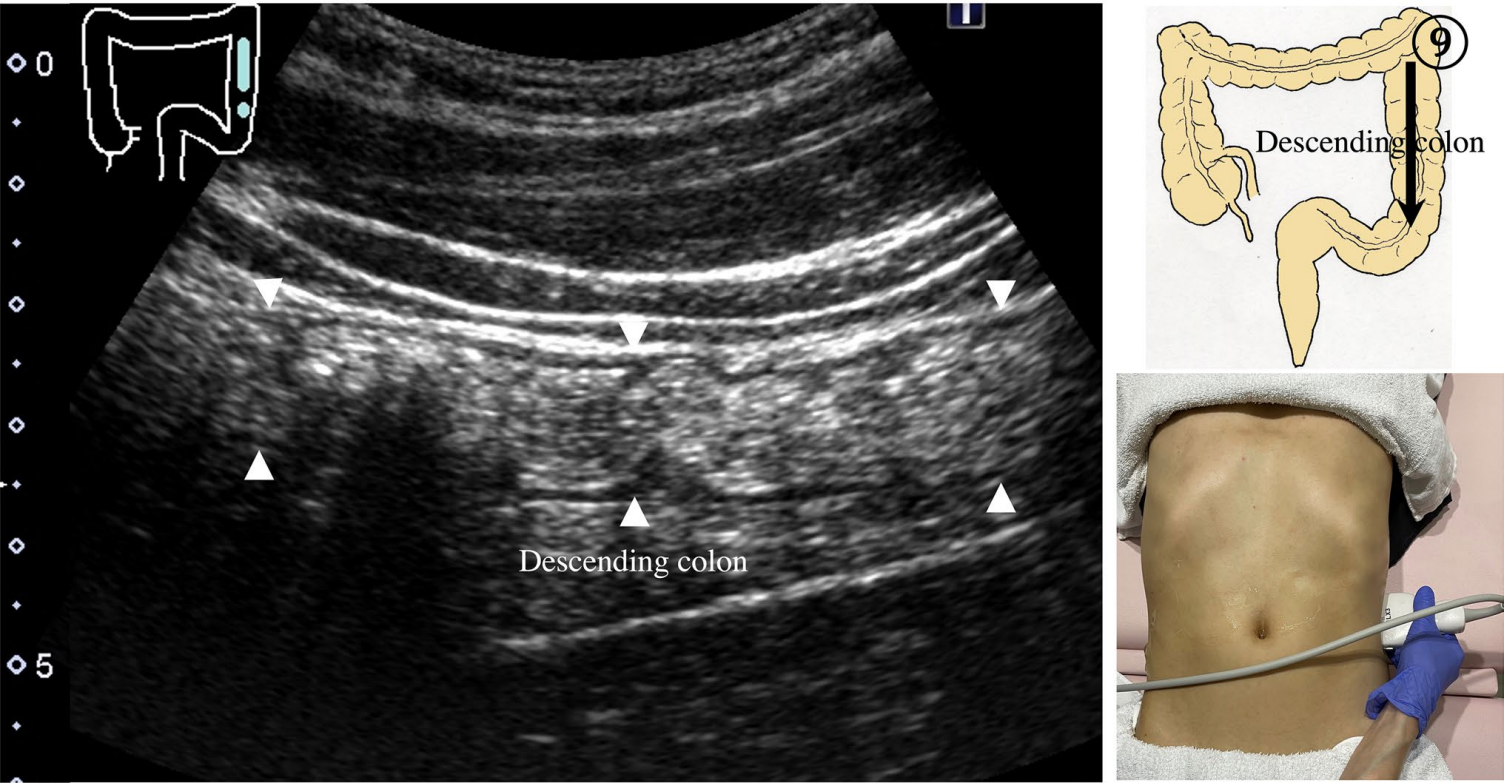
**Descending colon** Longitudinal view of the descending colon in the outermost and backmost region of the abdominal cavity

**Fig. 22:**
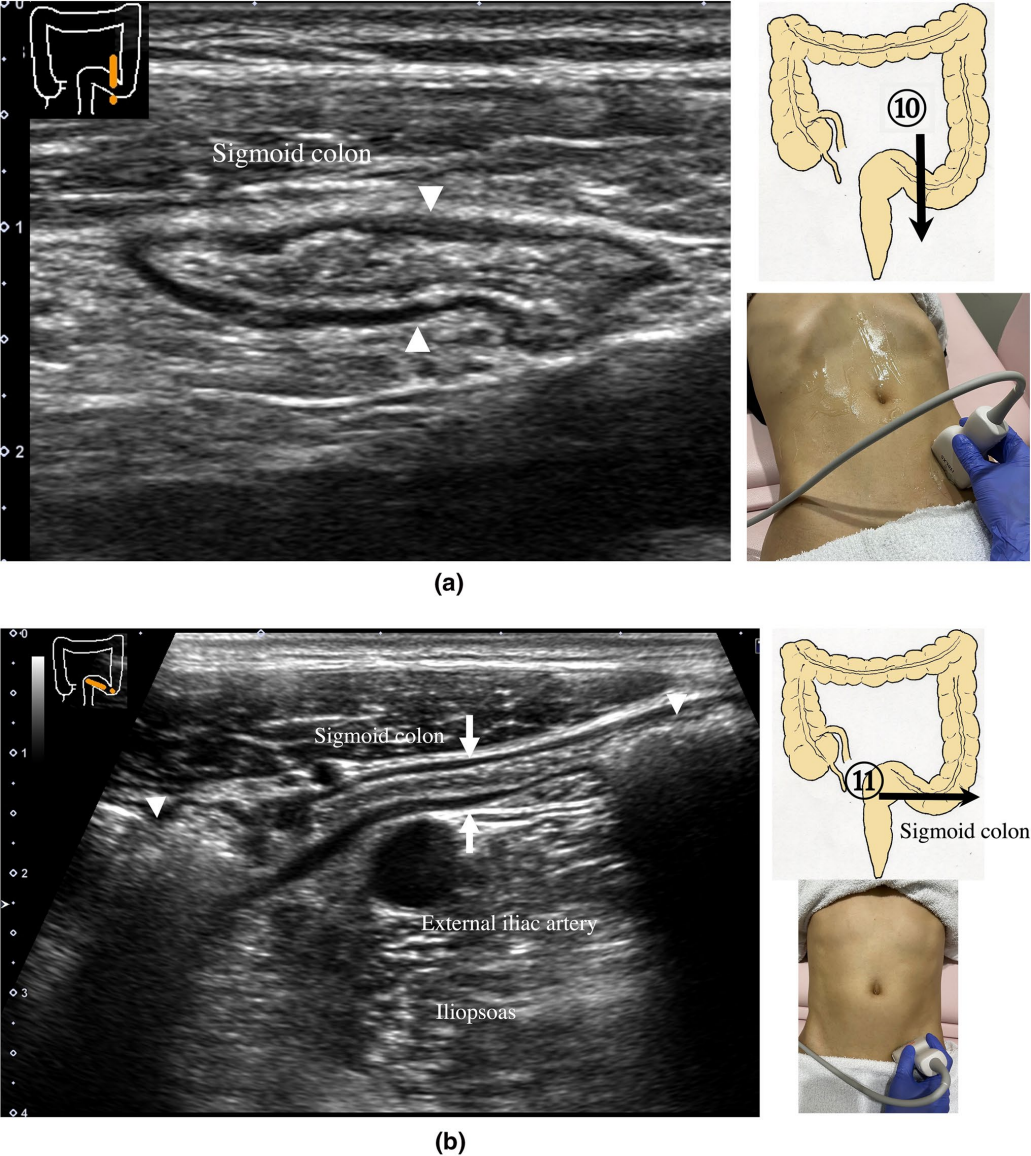
**Sigmoid colon** Short-axis view of the sigmoid colon (**a**). Longitudinal view of the sigmoid colon in front of the iliopsoas muscle and the external iliac artery (**b**). The central part is partially collapsed (arrow), while the oral and anal sides contain a relatively hard stool with a distinctive acoustic shadow (arrowheads)

**Fig. 23:**
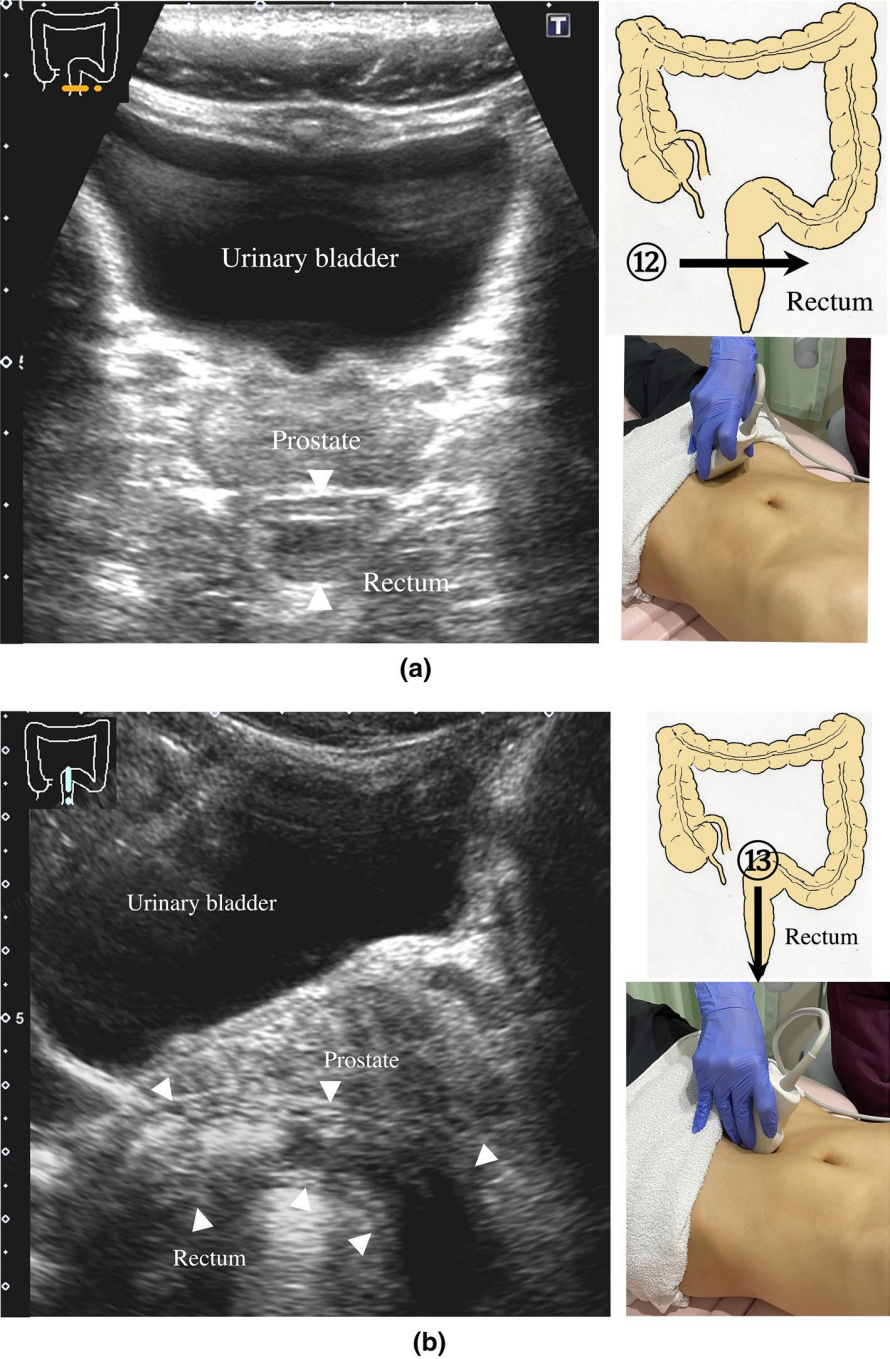
**Rectum** A view of the entire rectum (RS-Ra) is usually difficult to obtain. Short-axis view of the rectum (Rb) (**a**). Longitudinal view of the rectum (Rb) through the urinary bladder behind the prostate on the midline of the lower abdomen (arrowhead) (**b**)

9. The scan starts by identifying the ascending colon on an axial plane (Fig. 18a); should be in the most lateral and posterior position in the abdominal cavity. Then, the probe is turned to the sagittal plane, revealing haustra of the ascending colon (Fig. 18b). Scanning proceeds toward the cecum, which has a blind end. Attention is needed for thin patients as sometimes the cecum is in the pelvic cavity.
10. The appendix orifice is identified 1–2 cm caudal and ipsilateral to Bauhin’s valve [59, 60]. To identify the appendix, the probe is placed on the axial plane and moved approximately 5 cm cranially, where switching to a high-frequency probe (more than 7.5 MHz) is recommended. Scanning from cranial to caudal shows the ascending colon on half of the US monitor, while the terminal ileum continues vertically to Bauhin’s valve, viewed as a “mushroom sign” from the left side, with peristalsis. The terminal ileum runs in front of the iliopsoas and iliac vein and artery. In contrast, the appendix is observed as a smooth continuous structure connected to the cecum; from an ipsilateral connection of the ileum, it appears as a “beak sign” 1–2 cm caudal to Bauhin’s valve, with a blind end and no peristalsis (Fig. 19). The appendix is attached to the mesoappendix, which results in high morbidity and is found in various locations. Therefore, careful attention is needed. In most cases, the appendix runs in front of the iliopsoas muscle toward the pelvic cavity. If it cannot be identified, the pelvic cavity needs to be observed behind or outside of the cecum. In approximately 10% of the population, the appendix is found in a retrocecal location.
11. The examination proceeds to the sagittal plane on the midline to identify the transverse colon located on the caudal side of the gastric antrum (Fig. 20a). The probe is rotated at 90° to trace the transverse colon to the hepatic flexure (HF) and splenic flexure (SPF) (Fig. 20b). Under deep inhalation, the HF and SPF move caudally away from the ribs. If there is difficulty identifying the HF or SPF even under deep inhalation, the left or right decubitus position can be effective.
12. The descending colon is located in the most lateral and posterior region of the left side of the abdominal cavity and should be identified by a combination of axial and sagittal scans (Fig. 21). The angle of approach for the probe is shown in Fig. 17.
13. Finally, the colon is traced from the sigmoid colon in front of the iliopsoas muscle (Fig. 22) to the rectum, which is visualized through the urinary bladder (Fig. 23). The sigmoid to rectosigmoid colon is usually difficult to trace completely because the length and course vary, and intestinal gas may interfere with their observation in the pelvic cavity. The rectum is located dorsal to the prostate in males and the uterus in females.

During the scan of the colon and intestine, the graded compression method is used [61, 62], following the respiratory movements. When the patient exhales, the probe is gradually compressed to keep the target in focus. This compression can push away gas in the bowel and intraabdominal fat. This technique enables deeper areas to be reached with a high-frequency probe such that high-resolution images can be obtained, especially in the pelvis.

## GI wall layers and wall thickness cutoff value

The thickest and widest parts of each segment are measured preferably at the center part of the probe to determine the thickness of the wall and dilatation of the tract, respectively. Abnormal thickening of the GI tract wall is defined as a thickness greater than 5 mm in the stomach and rectum and greater than 3.5 mm in the small intestine and colon [45, 45, 46, 63–65]. Dilatation of the small intestine is defined as a diameter greater than 18 mm when filled with fluid. The appendix is less than 7 mm in diameter in adults [30, 59, 60, 62, 66–68]. The wall thickness changes according to the degree of dilatation and constriction of the intestine, as well as the location, such as the stomach and the rectum. Additionally, differences in wall thickness depending on age, sex, and food intake have been reported.

**Fig. 24:**
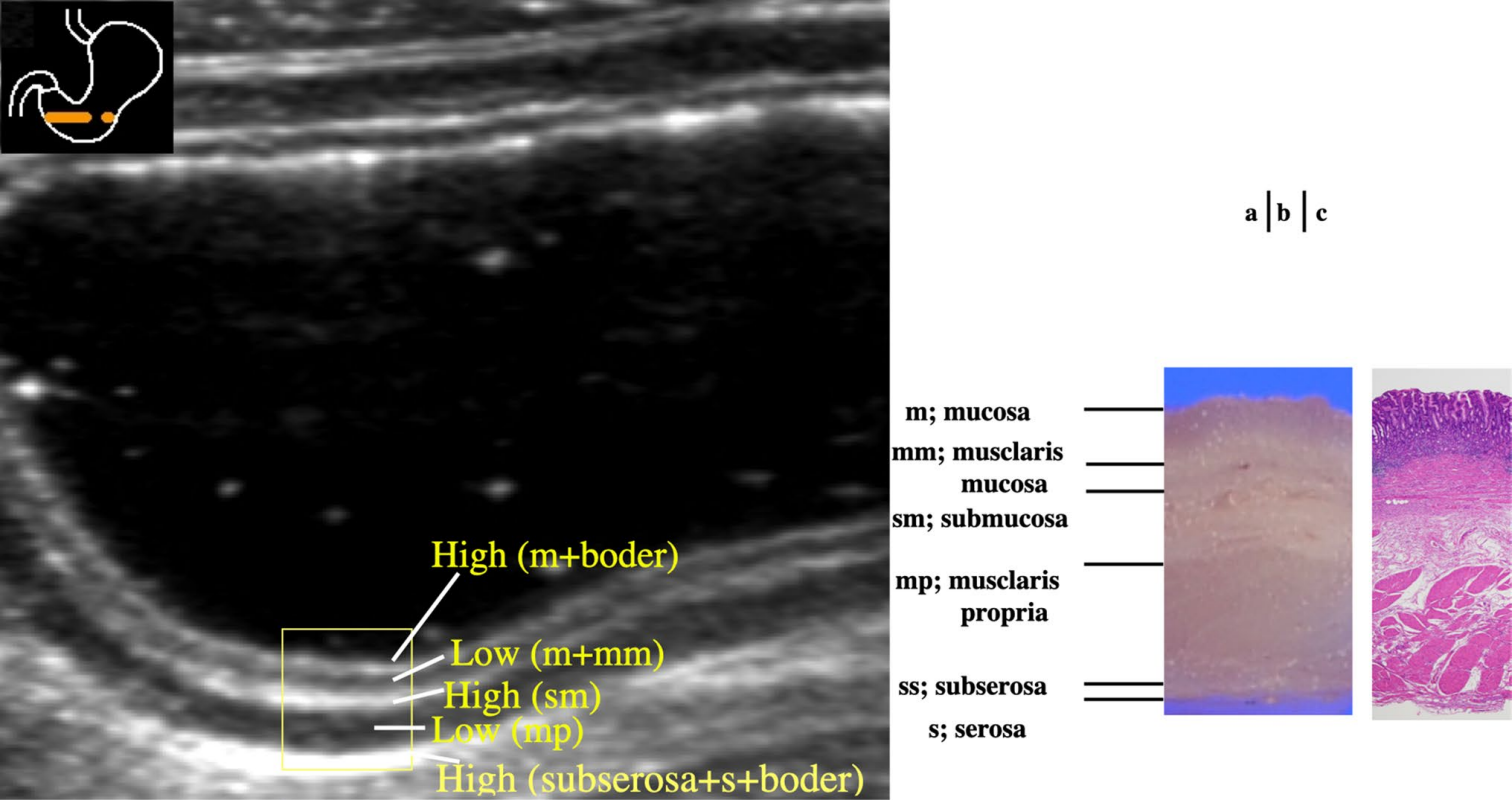
**Wall layer structure** US of the normal gastric antrum [65] (**a**). Five layers are visible in the normal stomach (rectangle). The first layer corresponds to the border echo and a superficial mucosal layer; the second layer corresponds to the rest of the mucosa; the third layer corresponds to the submucosa, an acoustic interface between the submucosa and muscularis propria; the fourth layer corresponds to the rest of the muscularis propria; and the fifth layer corresponds to the serosa and subserosal fat. Macroscopic specimen of the gastric antrum (**b**). Histological findings of the gastric antrum (HE stain) (**c**). *m* mucosa, *mm* muscularis mucosa, *sm* submucosa, *mp* muscularis propria, *ss* subserosa, *s* serosa

US detects five layers of the normal GI tract wall (Table 1) [47, 69]. Five layers are also visible in the normal stomach (Fig. 24). From the inside to the outside of the intestine, layers with high, low, high, low, and high echogenicity are observed. The first layer corresponds to the border echo and mucosa; second layer, mucosa and muscularis mucosa; third layer, submucosa; fourth layer, muscularis propria; and fifth layer, serosa and border echo (Table 1).

**Table 1:** Relationship between US and histopathological specimens

Wall layers identified by US	Histopathological specimen
1st layer; High echo	Border + mucosa
2nd layer; Low echo	Mucosa
3rd layer; High echo	Submucosa
4th layer; Low echo	Muscularis propria
5th layer; High echo	Subserosa + serosa

## Ten key parameters for diagnosing GI disorders

Diagnoses can be considered according to the following 10 key parameters (Table 2).

- ① Location: anatomical location identified by systematic scanning [40, 70]; distribution: diffuse (lesion continuity) or localized [71]
- ② Wall thickness: thickness of the wall of the lesion measured at the thickest part
- ③ Wall layer structure: clear, unclear, or disrupted (low echo in the submucosal layer)
- ④ Echo level: compared to each normal layer [72]
- ⑤ Wall deformation: ulceration or infiltration outside the wall
- ⑥ Dilatation: stenosis of the intestinal lumen observed by tracing dilated bowel
- ⑦ Wall stiffness: variability and compliance
- ⑧ Peristalsis: careful attention is needed to avoid morbidity
- ⑨ Findings outside the wall: lymph node enlargement, existing ascites, highly echogenic thickened mesentery/omentum/retroperitoneal fat
- ⑩ Blood flow signal: active inflammation/neovascularization suggested by increased blood flow signal [41, 44, 73–78]

**Table 2:** Ten key parameters for diagnosing gastrointestinal diseases with ultrasonography

#	Parameters
1	Location/distribution (diffuse/localized)
2	Wall thickness (mm)
3	Wall layer structure
4	Echo level [72]
5	Wall deformation
6	Dilatation
7	Wall stiffness
8	Peristalsis
9	Findings outside the wall
10	Blood flow signal

## Clinical utility

US can serve as a first-choice modality in screening for GI diseases. Especially in cases of acute abdomen, such as appendicitis, diverticulitis, and bowel obstruction, US is a useful tool to identify the lesion and confirm the diagnosis. Additionally, US can be used to monitor inflammatory bowel diseases and evaluate disease activity and complications. In particular, color Doppler is useful for monitoring disease activity in the same patient with the same parameter settings.

Detailed US examinations can be performed with high-frequency probes in cases of submucosal tumors and malignant tumors.

Typical GI disorders that can be diagnosed by US are shown in Table 3.

**Table 3:** GI diseases useful for US diagnosis

1	Esophageal/gastric/small intestinal/colonic cancer [13, 22, 53, 79–85]
2	Malignant lymphoma [35, 86]
3	Submucosal tumor [35, 64, 87–91]
4	Acute gastric mucosal lesion [12, 13]
5	Peptic ulcer [92, 93]s
6	Hypertrophic pyloric stenosis [22, 94]
7	Crohn’s disease [15, 25, 38, 57, 95–112]
8	Ulcerative colitis [15, 25, 38, 58, 102, 104, 113–116]
9	Ischemic colitis [22, 43, 117]
10	Bacterial enteritis [22, 118, 119]
11	Antibiotic-associated hemorrhagic colitis [120, 121]
12	Pseudomembranous colitis [22, 122]
